# Sample Digestion and Combined Preconcentration Methods for the Determination of Ultra-Low Gold Levels in Rocks

**DOI:** 10.3390/molecules24091778

**Published:** 2019-05-08

**Authors:** Yan-hong Liu, Bo Wan, Ding-shuai Xue

**Affiliations:** State Key Laboratory of Lithospheric Evolution, Institute of Geology and Geophysics, Institutions of Earth Science, Chinese Academy of Sciences, Beijing 100029, China; liuyanhong@mail.iggcas.ac.cn (Y.-h.L.); xuedingshuai@mail.iggcas.ac.cn (D.-s.X.)

**Keywords:** gold, sample preparation, preconcentration, geological samples

## Abstract

The gold abundance in basic rocks, which normally varies between 0.5 and 5 ppb, has served as a very important indicator in many geoscience studies, including those focused on the planetary differentiation, redistribution of elements during the crustal process, and ore genesis. However, because gold is a monoisotopic element that exhibits a nugget effect, it is very difficult to quantify its ultra-low levels in rocks, which significantly limits our understanding of the origin of gold and its circulation between the Earth crust, mantle, and core. In this work, we summarize various sample digestion and combined preconcentration methods for the determination of gold amounts in rocks. They include fire assay, fire assay combined with Te coprecipitation and instrumental neutron activation analysis (INAA) or laser ablation inductively coupled plasma mass spectrometry, fusion combined with Te coprecipitation and anion exchange resins, dry chlorination, wet acid digestion combined with precipitation, ion exchange resins, solvent extraction, polyurethane foam, extraction chromatography, novel solid adsorbents, and direct determination by INAA. In addition, the faced challenges and future perspectives in this field are discussed.

## 1. Introduction

**Figure 1:**
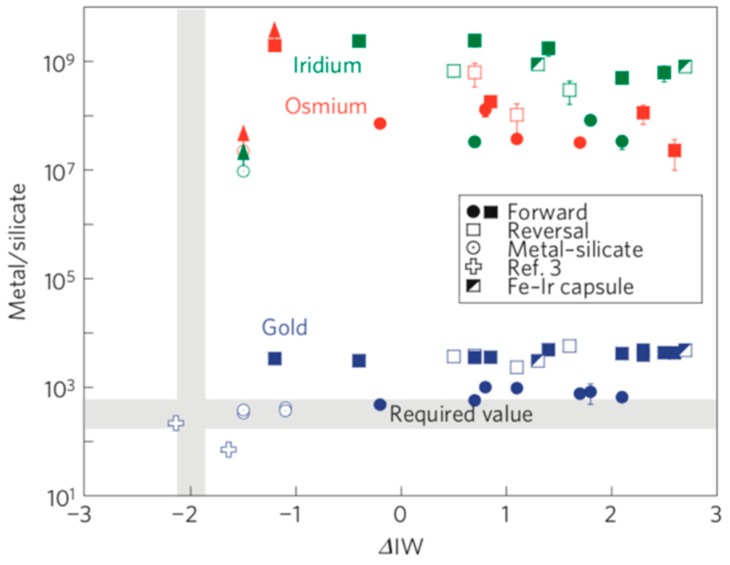
Metal-silicate partition coefficients as functions of the oxygen fugacity. Reprinted with permission from [10].

**Figure 2:**
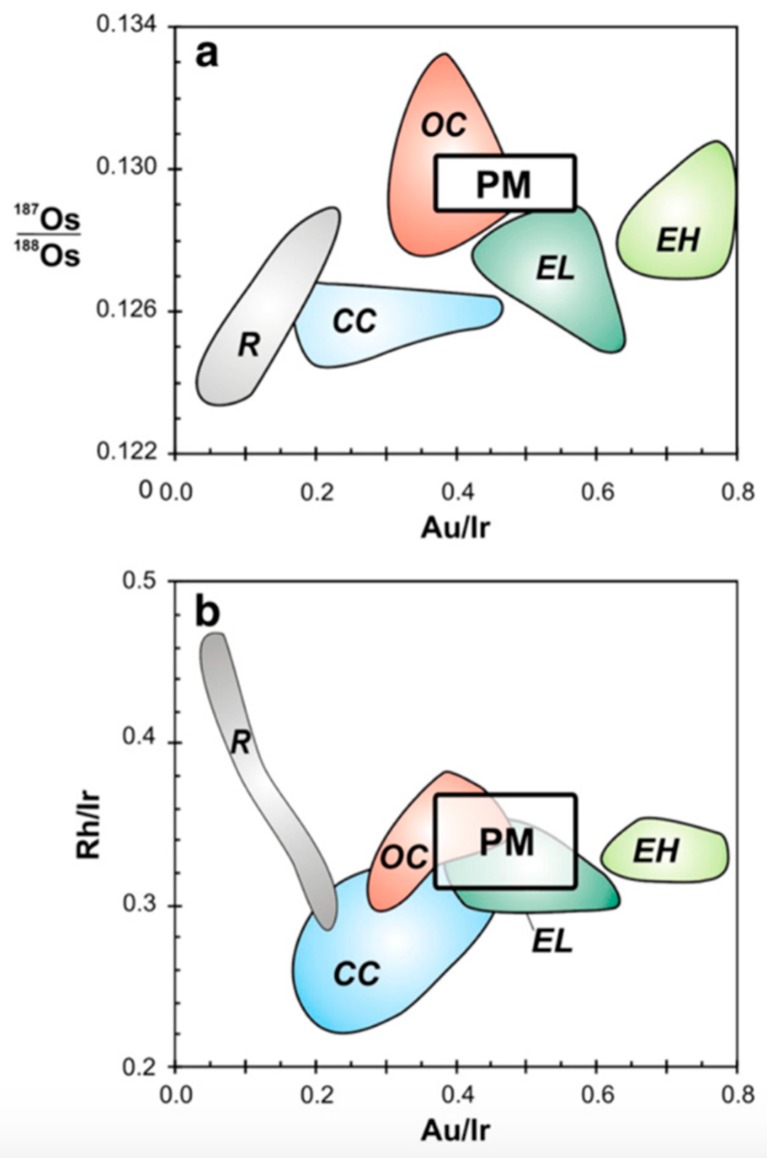
Ratio diagrams of Au/Ir vs. ^187^Os/^188^Os and Au/Ir vs. Rh/Ir constructed for the PM and different chondrite classes and groups. The HSE composition determined for the PM model is similar to those of ordinary (OC) and enstatite (EL) chondrites, but differs from the compositions of carbonaceous (CC) and Rumuruti (R) chondrites. Reprinted with permission from [5].

Gold is one of the rare elements and precious metals present in the Earth crust with average concentrations in the igneous, sedimentary, and metamorphic rocks varying between 0.5 and 5 ppb [1]. Proper quantification of the gold abundances in basic rocks is critical for many leading-edge areas of geoscience, such as planetary differentiation [2,3,4,5,6,7,8,9,10], redistribution of elements during crustal processes [11,12,13,14,15,16,17], and ore genesis [18,19,20,21,22,23]. According to the latest results reported by Brenan and McDonough [10], the metal–silicate partition coefficient of Au is approximately 300, whereas its minimum values measured for Os and Ir in the same experiments are ~10^7^, which differs from the former parameter by at least a factor of 10^4^. The authors concluded that not all highly siderophile elements (HSEs) were affected by the core formation in the same way, and that the abundances of elements such as osmium and iridium required the addition of a late veneer. As an illustration, Figure 1 shows the metal-silicate partition coefficients plotted as functions of the oxygen fugacity. Fischer-Gödde et al. analyzed samples of orogenic peridotite massifs and xenoliths, whose Rh and Au contents revealed the presence of HSEs in the primitive mantle (PM), which differed from that of any known group of chondrites and could be explained by the contributions from meteoritic components detected in ancient lunar impact melt rocks [5]. Figure 2 shows the ratio diagrams of Au/Ir vs. ^187^Os/^188^Os and Au/Ir vs. Rh/Ir constructed for the HSEs of the PM and different chondrite classes and groups. In addition, as no explorations conducted during the past 50 years detected deep gold deposits in Earth’s crust, the world is thought to be tottering on the precipice of peak gold [24]. As a result, it is very important to discover large deposits of gold and meet the production demand in the field of gold ore genesis. However, the precise determination of ultra-low gold contents in rocks is an extremely difficult task as compared with detecting other trace elements because gold represents a monoisotopic element and exhibits a nugget effect. The latter requires the analysis of relatively large amounts of rocks to obtain meaningful data, which are still characterized by large deviations. Since gold has only one isotope (^197^Au), it cannot be quantified by an isotope dilution (ID) method that is very precise for trace element determination and, therefore, requires a simple analytical method for its quantitative recovery during pretreatment.

Although modern analytical techniques such as inductively-coupled plasma mass spectrometry (ICP-MS), graphite furnace atomic absorption spectroscopy (GFAAS), and instrumental neutron activation analysis (INAA) are highly sensitive, their use for the direct determination of gold levels in geological samples is complicated because of the low concentrations of gold and interfering effects of matrix components. To increase the reliability of gold quantitation methods, the major matrix components must be separated first, which can be achieved by proper sample preparation and the preconcentration of gold [25,26,27,28].

Sample digestion and/or preconcentration technologies used for the determination of gold as well as platinum group metals (PGEs) with ppm concentrations were previously reviewed by Perry, Barefoot, Balcerzak, Pyrzynska, Mokhodoeva, Myasoedova, and their co-authors [25,29,30,31,32,33,34,35]; however, methods for the detection of ultra-low gold levels in rocks (which are considerably more complex than ore-grade samples) have not been discussed in detail. This review includes the recent significant contributions to the determination of gold amounts in rocks, especially those focused on sample preparation and the preconcentration of gold prior to its determination. As of today, almost all distributions and parameters of gold in geological samples have been determined by fire assay. To validate them and obtain more data, independent methods such as diisobutyl ketone (DIBK) extraction chromatography and standardization combined with ID ICP-MS cation exchange resin analysis have been developed [5,36]. However, due to the long procedure or expensive spiking, a simple and reliable technique for measuring the contents of gold in rocks must be used. In this work, we describe various sample digestion methods that are often coupled with enrichment techniques and discuss their mutual effects.

## 2. Dry Digestion Methods

### 2.1. Fire Assay

Fire assay (FA) and cupellation methods are classical assaying techniques that have been successfully used for the estimation of gold amounts in ores for many centuries [37]. Furthermore, FA has always served as the arbitration method of gold measurement that involves not only the digestion of samples, but also the enrichment of gold and PGEs since it allows the extraction of these precious metals and their separation from base metals in the matrices. The relatively large sample weights used for FA can overcome the nugget effect, which represents its significant advantage. However, the large reagent blanks that result from the large amounts of fluxes introduce significant biases into the determination of ultra-low gold levels. In addition, the uncertainty of the chemical interactions between various flux constituents makes the quality of the obtained results highly dependable on the experience of the analyst.

Lead fire assay (Pb-FA) and nickel sulfide fire assay (NiS-FA) are widely used FA methods. Pb-FA utilized for the collection of Au, Pt, Pd, and Rh is the application of metallurgy in analysis. A simple Pb-FA method has been developed in 1994 for the determination of ultra-low Au, Pt, and Pd contents in rocks by Hall et al. [38]; its limit of detection (LOD) and recovery of gold are 2 ppb and 90%, respectively. The large relative standard deviations (RSD) of 11–55% are likely caused by the sample inhomogeneity rather than by the analytical method. This conclusion is in good agreement with the recoveries of gold ranging from 74–86% at concentrations between 30 and 300 ppb [39]. NiS-FA can be used to collect both PGEs and gold, but its efficiency for gold determination has been very low. Juvonen et al. [40] compared NiS-FA with Pb-FA in terms of their collection efficiencies of Au, Pt, Pd, and Rh and found that the gold recovery of NiS-FA was twice as low as that of Pb-FA at low gold concentrations. Plessen and Erzinger [41] reported that the gold recovery did not exceed 70% when NiS-FA was used to analyze rocks. Although the gold recovery of Pb-FA is better than that of NiS-FA, the environmental pollution and harm to analysts caused by Pb discouraged its further development [42,43]. However, the bottleneck of the analysis of the low gold contents in rocks by NiS-FA consists of the high amounts of reagent blanks and low recovery efficiency of gold.

Many researchers have attempted to solve this problem. In order to lower the reagent blank, Asif and Parry [44] prepared a mini button by reducing the amount of nickel reagent; however, the recovery of gold exhibited a significant reduction. Lu et al. [45] found that FA combined with a Te coprecipitation purification process could lower the NiO blank to 0.24 ppb. In terms of the gold recovery, two specific directions exist: combining NiS-FA with Te coprecipitation to reduce the loss during the acid dissolution process and employing a solid direct determination technology (such as INAA and laser ablation inductively coupled plasma mass spectrometry (LA-ICPMS)) to reduce the loss and possible pollution in each process following the FA step.

#### 2.1.1. NiS-FA + Te Coprecipitation

As large amounts of gold and small PGEs contents are lost in the acid solution during the dissolution of the NiS button, a second enrichment step that would not increase the amount of total dissolved solids must be introduced to improve the gold recovery (especially at low concentrations). Jackson et al. [46] applied the Te coprecipitation method to gold analysis for the first time and increased the recovery of gold from ore samples to 90%. Savard et al. [47] obtained the same results for ore samples, but the recovery remained low for rock samples. In addition, although the sample weight in this method was as large as 15 g, the RSD amounted to 20–50% (the reason for this phenomenon is not clearly understood). Oguri et al. [48] obtained high recoveries (>97%) for low gold concentrations by repeating the NiS-FA procedure under the reduced conditions (produced by graphite powders) and Te coprecipitation at the optimal conditions corresponding to a temperature of 210 °C and time of 75 min. In order to lower the reagent blank and simplify the process, a semi-open NiS dissolution system preventing volatile losses was suggested by Gros et al. [49]. In addition, Sun and Sun [50] proposed a novel NiS-FA method involving Fe, which ensured the self-disintegration of the entire assay button into powder without its mechanical crush. When some particular samples are used (such as black shale or samples containing magnetite), conventional NiS-FA is not applicable. Li [51] ignited black shale samples before the NiS-FA step to eliminate organic matter. Juvonen et al. [52] used potassium tartrate as a reducing agent to successfully prepare an assay button during the analysis of samples containing magnetite.

#### 2.1.2. FA + INAA or LA-ICPMS

**Figure 3:**
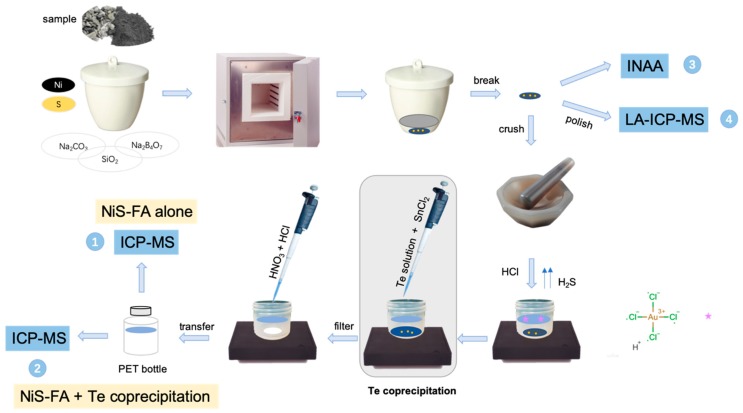
Main stages of the gold determination by the NiS-FA method.

After a method that used only 0.5 g of nickel was developed (which decreased the button weight to below 1 g), it enabled the direct analysis of small gold-containing buttons by INAA or LA-ICPMS [44]. Asif et al. [53] proposed a simple method based on NiS-FA and INAA to determine the levels of PGEs and gold in samples. However, the LOD of gold was 2 ppb, which made this technique unsuitable for ultra-low gold contents. Bedard and Barnes [54] compared the capacities of FA-ICP-MS and FA-INAA to determine the gold amounts in geological samples and found that for the specimens rich in gold both methods performed adequately; however, for the low-concentration samples (crustal rocks), ICP-MS was preferable. Jarvis et al. [55] established for the first time a method for gold quantitation based on the combination of NiS-FA with LA-ICP-MS having an LOD of 10 ppb. An ultraviolet (UV) laser ablation ICP-MS was used to directly analyze the gold contents in NiS-FA buttons by Jorge et al. [56]. The obtained LOD of gold was as low as 1.7 ppb, and the RSDs were better than 10%. Later, Resano et al. [57,58] ground NiS buttons to obtain more homogeneous samples and used polyethylene wax as a binder to pelletize the resulting powders (possible interferences were eliminated by utilizing a double-focusing sector field mass spectrometer). Resano et al. [59], Vanhaecke et al. [60], and Compernolle et al. [61] discussed the possibility of combining Pb-FA with LA-ICPMS for the analysis of gold in geological samples. Meanwhile, Compernolle et al. [61] reported the absence of significant differences between the results obtained by the standard addition, internal standard, and external standard methods using Pb-FA with LA-ICPMS for gold determination. Table 1 summarizes various FA digestion methods used in literature studies. Figure 3 illustrates the main stages of the four techniques for the gold determination by the NiS-FA method described in several works. Here, number 1 denotes the method that combines NiS-FA alone with ICP-MS according to Plessen and Erzinger [41]. The polyethylene terephthalate (PET) bottle is used to store the gold solution. Number 2 denotes the technique that combines the NiS-FA method with Te coprecipitation for gold extraction based on the works of Jackson et al. and Savard et al. [46,47]. Simpler methods that combine NiS-FA with INAA or LA-ICP-MS are represented by numbers 3 and 4, respectively. They are based on the approaches developed by Asif et al. and Jorge et al. [53,56].

### 2.2. Fusion

Fusion is an effective sample decomposition method that is mainly used to dissolve acid-insoluble substances. In the process of fusion, insoluble samples are converted into sodium compounds that are soluble in water or acids. Fusion is very different from FA, although both techniques are dry digestion methods. In particular, fusion is just a sample digestion method, whereas FA involves both sample digestion and gold enrichment, and its ingredients and process are more complicated. Fusion is rarely used to decompose samples for the determination of gold contents; for this purpose, a sodium peroxide method is most commonly used. The greatest advantages of this technology include the ability to effectively decompose sulfide and refractory minerals and a large sample mass (up to 20 g). However, large contents of sodium peroxide with impurities are difficult to purify, and the contaminations resulting from the interaction of the flux with the crucible wall can negatively affect the course of analysis [62]. Another disadvantage of this method is the formation of gel-like soluble alkaline silicates during fusion that make the solution difficult to analyze.

Due to the absence of gold enrichment, fusion buttons are not suitable for direct testing (such as FA), but must be dissolved and treated by other enrichment techniques. The most common combination technologies utilize ion exchange resins [63,64] and Te co-precipitation [65,66,67]. A summary of various fusion digestion methods is presented in Table 2.

#### 2.2.1. Fusion + Anion Exchange Resin

Enzweiler [63] studied the recovery efficiencies of Pt, Pd, and Au from silicate rocks using a sodium peroxide fusion procedure followed by anion exchange resin separation. The utilized technique was found to be not very efficient: the recovery of gold was as low as 76% due to the formation of hydroxychloro compounds in alkaline solution that were not converted to chloro complexes upon the acidification with HCl that was required for quantitative anion exchange. Later, Dai et al. [64] combined an extra chlorination step with this technique to oxidize PGEs and gold and thus achieve the best retention in the anion resin separation process. However, no valuable data were obtained for gold because of the poor linear regression correlation of the external calibration curve for ICP-MS analysis.

#### 2.2.2. Fusion + Te Coprecipitation

Amosse [65] described a method for the extraction of Pd, Pt, Rh, Ru, Ir, and Au using Se and Te as carriers in the presence of catalyst KI after the fusion with sodium peroxide, sodium potassium carbonate, and potassium hydroxide. The catalyst improved the recovery of Ir from 33% to 97.5%, although it had no effect on the Au recovery efficiency. In order to simply this process, Jin and Zhu [66] used sodium peroxide fusion and Te coprecipitation to measure the PGE and Au levels in geological matrices. The procedural reagent blanks and recovery of Au were 0.044 ppb and 80%, respectively. Qi et al. [67] purified HCl and SnCl_2_ by Te coprecipitation to further lower the reagent blanks and achieved a good Au recovery of 96.3%.

#### 2.2.3. Fusion of the Residue Formed after Acid Digestion

Another important application of fusion is the ability to recover all gold from the sample residue after acid digestion. Totland et al. [68] used the Na_2_O_2_ + Na_2_CO_3_ mixture and pure Na_2_O_2_ to dissolve the sample residue formed after the HNO_3_ + HCl + HF + HClO_4_ microwave digestion process. Jarvis et al. [69], Tsimbalist et al. [70], and Coedo et al. [71] employed the same method to treat the acid-insoluble residue formed after the HNO_3_ + HCl + HF acid digestion. The insoluble residue of black shale samples produced after BrF_3_ digestion was melted by KBrF_4_ [72].

### 2.3. Dry Chlorination

Chlorine has been the most important gold leaching agent from 1850 to 1900. Afterwards, because of the higher selectivity and lower price of cyanide, the latter gradually replaced chlorine in the gold leaching of sulfide ore. Since that time, little research on the chlorination of gold has been conducted. The advantages of chlorination include very low procedural reagent blanks, high analytical efficiency, and large sample weight. However, the low recovery of gold is a drawback of this method. Nesbitt et al. [73] studied the chlorination mechanism of gold in aqueous solutions and found that the gold particle size was the main factor affecting it. Perry and Van Loon [74] utilized the chlorination process for the determination of ultra-low gold contents in rocks for the first time. A gold recovery of 60% was achieved under the following optimal conditions: 3.5 h, 580 °C, 0.5 g NaCl, and 15 g sample powder. Although the recovery was relatively low, the sensitivity and precision of this technique were comparable or better than those of the FA method. In order to further improve the data precision, Perry et al. [75] attempted to increase the sample weight to 250 g to reduce the nugget effect. Furthermore, chlorination was also used to oxidize PGEs and gold to higher oxidation states that were more strongly absorbed by anion resins [64].

## 3. Wet Acid Digestion Methods

Wet acid digestion is a widely used sample preparation technique. It is considered an alternative to FA for the extraction of PGEs and gold from geological samples. This method has many advantages, such as simplicity, high speed, low cost, robustness, simple reagent ingredients, low blank, and high degree of sample universality. However, the sample weight used during acid treatment is usually below 10 g, which is smaller than that of the dry digestion methods; as a result, the sampling errors obtained by this technique are large due to the stronger nugget effect. In addition, its efficiency depends on the ratio of the sample weight to the acid volume.

**Figure 4:**
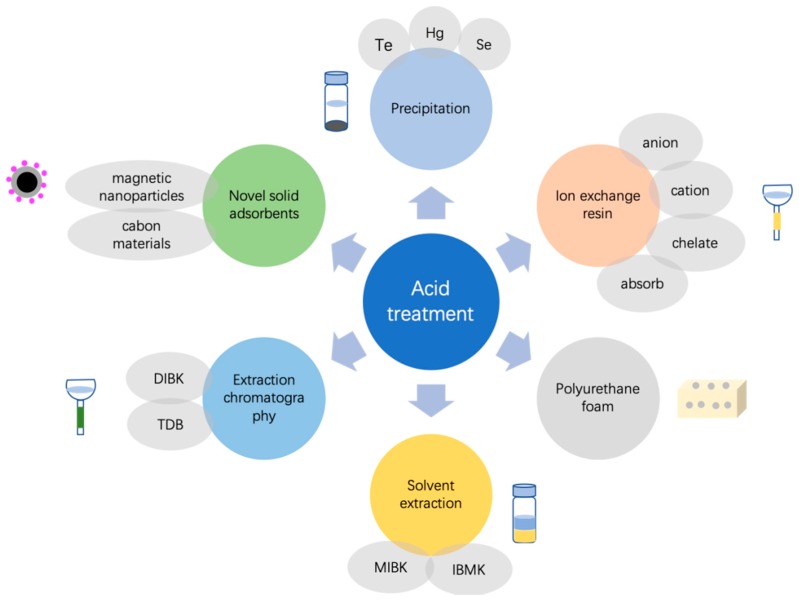
Common preconcentration methods used with wet acid digestion.

The best acid for gold extraction is aqua regia (aq. reg.) that can dissolve compounds insoluble in HCl or HNO_3_ alone. The addition of HF, NH_4_F, Br_2_, KClO_3_, or H_2_O_2_ significantly increases its strength. HF or NH_4_F can react with SiO_2_ to form SiF_4_ and destroy the silicate structure, which facilitates the quantificational extraction of gold from geological samples. In many studies [37,39,76,77,78,79], it was found that HSEs could not be quantifiably extracted from rocks without desilication. Br_2_, KClO_3_, or H_2_O_2_ can oxide HCl to produce more chlorine gas, which increases the gold solubility. Normally, an additional preconcentration step must be performed after acid digestion during the analysis of ultra-low gold contents in rocks. The commonly used enrichment methods use precipitation [26,80,81,82,83], ion exchange resins [5,71,81,84,85,86,87,88], solvent extraction [27,89,90,91,92,93,94,95,96,97,98], polyurethane foam (PUF) [99,100,101,102,103,104,105,106,107,108,109,110,111,112,113,114,115,116,117,118,119,120,121,122,123,124,125,126,127,128,129,130], extraction chromatography [36,131,132,133], and solid adsorbents [134,135,136,137]. Figure 4 shows the common preconcentration methods utilized with wet acid digestion.

### 3.1. Acid Digestion + Precipitation

A method combining acid digestion with Te coprecipitation was originally used in 1978 to measure the amounts of gold and silver in rocks [80]. Later, Gupta [81] pointed out that Te interfered with the determination of gold and silver during the atomization step; therefore, this method was not suitable for quantifying gold and silver, but could be used to measure the contents of other PGEs instead. In order to prevent the loss of gold during the dissolution of tellurium buttons, acid digestion, Te coprecipitation, and INNA were utilized to determine the gold contents in rocks [82]. The gold recovery and LOD of this method were 96% and 0.7 ppb, respectively. Eller et al. [26] used a polytetrafluoroethylene (PTFE) pressure bomb combined with HNO_3_, HF, aqua regia, and HClO_4_ to dissolve rock samples, while Se coprecipitation was performed to preconcentrate gold. The obtained recovery of Au was greater than 98%. Niskavaara and Kontas [83] combined HF and aqua regia acid digestion with Hg coprecipitation to determine the concentrations of Au, Pd, Pt, Rh, Ag, Te, and Se in geological samples. The observed poor precision of gold in the low-concentration samples was likely caused by sample heterogeneity. Table 3 lists various methods for Au preconcentration.

### 3.2. Acid Digestion + Ion Exchange Resin

• Anion exchange resins

Since gold exists in the form of AuCl_4_^−^ complex anions under acidic conditions, when an acidic solution containing AuCl_4_^−^ is passed through an anion exchange resin, AuCl_4_^−^ is adsorbed on the resin and becomes separated from other basic metals. However, it is difficult to elute the AuCl_4_^−^ species strongly adsorbed on the anion resin surface. Barredo and Polo [84] developed a method for the analysis of Au, Ag, and Cd in rock samples, which were digested with HF, aqua regia, and HClO_4_, preconcentrated with Dowex 1-X8 anion resin, and eluted with ammonia solution.

• Cation exchange resins

In contrast to the anion exchange resins, when an acidic solution containing AuCl_4_^−^ is passed through a cation exchange resin, gold is transported through the resin column in the form of AuCl_4_^-^ complex anions separated from metal cations. Gupta [81,85] compared the enrichment efficiencies of Dowex 50W-X8 cation exchange resin combined with Te coprecipitation for PGEs and gold. The cation exchange resin method is recommended for the determination of µg/g levels of gold. Based on the results reported by Meisel et al. [86] showing that all PGEs exhibit similar chemical behavior in a chromatographic column, they can be used as the ideal internal standards for the calculation of Rh concentrations. Fischer et al. [5] used a Carius tube filled with inverse aqua regia to dissolve rock samples and Eichrom 50W-X8 cation exchange resin to separate Re, Ir, Ru, Pt, Rh, Pd, and Au from the matrix. The concentrations of monoisotopic Rh and Au elements were calculated by the standardization to the ^101^Ru and ^193^Ir signal intensities, and the Ru and Ir concentrations were determined by isotope dilution.

• Chelating resins

Chelating resins contain chelating groups that can selectively adsorb gold and separate it from other matrix elements in solution. They combine the ionization exchange and complexation reactions and, therefore, exhibit good selectivity and strong binding energies as compared with those of the ordinary ion exchange resins. Coedo et al. [71] decomposed geological samples by aqua regia and HF and then separated PGEs and gold using tetraethylenepentamine chelating resin. Wu et al. [87] used YPA_4_ chelating resin as both the solid phase extractant and chemical modifier to determine the Au, Pd, and Pt contents in geological samples by electrothermal vaporization inductively coupled plasma atomic emission spectrometry (ETV-ICP-AES). The elution of gold from the two chelating resins mentioned above was performed by ashing.

• Chelate absorption resins

Chelate absorption resins combine the high selectivity of chelating resins with the high adsorption efficiency of absorption resins. Spheron Thiol 1000 chelate absorption resin was used to extract low gold levels by Medved et al. [88]. Table 4 summarizes the Au extraction methods using ion exchange resins.

### 3.3. Acid Digestion + Solvent Extraction/Dispersive Liquid–Liquid Microextraction

Solvent extraction (also called liquid-liquid extraction) is a classical method for Au separation and preconcentration characterized by the low reagent blank, high enrichment efficiency, and simple operation. The most widely used solvents for low-level Au determination are methyl isobutyl ketone (MIBK) and isobutyl methyl ketone (IBMK).

Terashima [89] established a simple method utilizing aqua regia for sample digestion and MIBK for gold extraction to determine gold concentrations in 60 geological reference materials. As aqua regia only partially interacted with the samples, Terashima et al. [90] adopted aqua regia and HF for dissolving the entire gold content. The obtained results were very close to those of INAA. Normally, the solvent extraction method is used in conjunction with GFAAS. However, the Fe spectral line of 242.4 nm can interfere with the sensitive Au line of 242.8 nm in the determination of Au by GFAAS. In addition, as the polarities of Au and Fe are close, they are always extracted together by the same solvent. Therefore, the elimination of iron impurities from the organic phase is the key to the accurate determination of low gold levels. A two-stage solvent extraction method (using diethyl ether and MIBK) was proposed by Yokohama et al. to prevent iron interference and effectively concentrate gold [27]. The results obtained for reference materials were in good agreement with the INAA data. Ramesh et al. [91] used the Zeeman background correction technology for GFAAS, washed the MIBK organic phase with a wash solution, and centrifuged it twice for the total removal of iron.

Chattopadhyay and Sahoo [92] used the sequential digestion of HBr-Br_2_ and aqua regia, sequential extraction of IBMK and toluene, and Te coprecipitation enrichment method to determine traces of gold in geological samples. Monteiro et al. [93] examined the stability of gold in IBMK extracts and found that closed polypropylene containers (with absorbance measurement changes of less than 3%) were more suitable than both the open polypropylene and closed glass containers over periods of up to 22 h.

As the liquid-liquid extraction method requires the use of large volumes of organic solvents (which are often toxic), Rezaee et al. [94] introduced a dispersive liquid–liquid microextraction (DLLME) method that was highly sensitive, efficient, and powerful for the preconcentration and determination of trace elements. Shamsipur and Ramezani [95] applied the DLLME technology to form an adduct between Victoria Blue dye and AuCl_4_^−^ using acetone as a dispersant and chlorobenzene as an extractant to determine the ultra-trace amounts of gold by GFAAS. Another DLLME method for the determination of gold traces using dicyclohexylamine as the extractant, acetone as the dispersant, and chloroform as the extraction solvent was established by Kagaya et al. [96]. Calle et al. [97] applied the DLLME technology to preconcentrate the ion pairs formed between AuCl_4_^−^ and [CH_3_(CH_2_)_3_]_4_N^+^ in a microliter-range volume of chlorobenzene using acetone as a disperser solvent for the determination of ultra-low gold contents. Fazelirad et al. [98] used benzyldimethyltetradecyl ammonium chloride dihydrate to form an ion pair with AuCl_4_^−^, acetone as the dispersant, and 1-hexyl-3-methylimidazolium hexafluorophosphate ([Hmim][PF_6_]) ionic solution as the extractant for gold extraction. Table 5 lists the solvent extraction/dispersive liquid-liquid microextraction methods for Au preconcentration.

### 3.4. Acid Digestion + PUF

**Figure 5:**
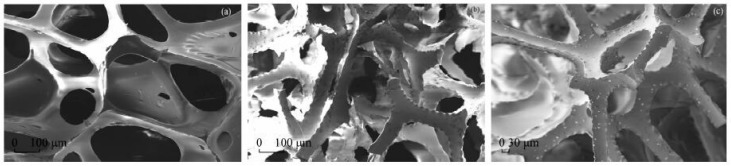
Scanning electron microscopy images of (**a**) PUF, (**b**) PUF-NH_2_, and (**c**) PUF-NH_2_ adsorbed Au. Reprinted with permission from [107].

PUF was originally used for the selective adsorption of gold from 0.2 M HCl solution in 1970 by Bowen [99]. After that, this technology has become widely spread, owing to its excellent adsorption selectivity, high enrichment efficiency, simple operation process, and low analysis cost [100,101]. The mechanism of PUF adsorption may be based on physical adsorption, solvent extraction, or ion exchange. However, the majority of studies support the solvent extraction-based mechanism. Gesser [102], Oren [103], Lo and Chow [104], and Jones et al. [105] examined the PUF adsorption of Ga, Fe, Sn, and Rh, and concluded that PUF was a “solid solvent-extractant”. In addition, a possible cation chelation mechanism was suggested by Hamon based on the results of his detailed studies on the PUF adsorption of Co thiocyanate and salts of several organic acids [106]. However, as shown by Wang et al. [107], the PUF adsorption of gold involved a reduction reaction, indicating that PUF reduced AuCl_4_^−^ (+3) to Au (0) followed by its deposition on the foam surface (Figure 5). It can be hypothesized that the mechanisms of PUF adsorption are not the same for different compounds.

PUF can be used as a special adsorbent for gold due to its very high selectivity. Schiller [108] compared the adsorption efficiencies of PbS, Fe(OH)_3_, Al(OH)_3_, Dowex 1-X8, and PUF toward gold. It was found that quantitative recovery of gold could be achieved within 90 min using PbS and PUF. However, the PUF method is superior to the PbS one in terms of the signal-to-noise ratio during the testing step. With the continuous optimization of various experimental conditions (including those for the pretreatment of PUF [109,110], types and concentrations of the acid digestion reagents and adsorption conditions [111,112,113,114], and elution conditions [115,116]), this method has become one of the main techniques for the enrichment of gold in geological samples [117,118,119,120,121]. To further improve the selectivity and adsorption capacity of PUF, many researchers coated it with organic reagents (such as MIBK and TBP) [122,123,124,125,126], bonded chelating ligands to the PUF matrix [127], and functionally modified it by the ligand coupling [128,129,130] with the PUF skeleton.

### 3.5. Acid Digestion + Extraction Chromatography

As extraction chromatography represents a chromatographic method, it exhibits the basic characteristics of chromatography. Unlike the commonly used chromatographic techniques, in this method, an inert carrier of organic extractant is supported on the column as the stationary phase for separation, and a solution of various inorganic acids is utilized as the mobile phase. This technology combines the high selectivity of extractants in solvent extraction with the effectiveness of chromatography separation, which significantly reduces the amounts of organic extractants and is less dangerous and easy to operate. In the 1970s, Pohlandt and Steele [131,132] and Bao [133] began to use porous silicon and polytrifluorochloroethylene as an inert carrier for tributyl phosphate extractant to analyze the gold amounts in ore samples and assay grains. Later, Pitcairn and Warwick [36] coated polyacrylamide resin with DIBK to preconcentrate ultra-low gold contents in rocks. The LOD of this method was as low as 0.002 ppb.

### 3.6. Acid Digestion + Novel Solid Adsorbents

Activated carbon enrichment is a widely used method for gold determination. This technique is very simple, and its separation effect is very strong during pulp application. However, the common elution method involves the direct ashing of activated carbon, which causes the loss of gold and contamination. To solve this problem, a new simple technology was developed by Hassan et al. [134], in which gold was adsorbed on granular activated carbon followed by graphite GFAAS analysis. Other carbon materials such as carbon nanotubes were also used for gold extraction. In order to improve its selectivity and adsorption capacity, Dobrowolski et al. [135] compared the effects of nitric acid, ethylenediamine, and (3-aminopropyl) triethoxysilane on the modification of carbon nanotubes. The efficiency of analysis can also be improved using a hybrid adsorbent. Xue et al. [136] developed an adsorbent composed of cellulose fibers, activated carbon, and anion exchange resin for the preconcentration and separation of Au, Pd, and Pt in geological samples. Furthermore, in recent years, magnetic nanoparticles have been widely used in sample extraction due to their unique magnetic response, large surface area, and chemically modifiable surface. Ye et al. [137] established an on-line method for the Au, Pd, and Pt determination with 4′-aminobenzo-15-crown-5-ether functionalized magnetic nanoparticles. The obtained LOD of gold was 0.16 ppb. Table 6 summarizes the Au preconcentration methods using PUF/extraction chromatography/novel solid absorbents discussed in this work.

## 4. Direct Determination Methods

INAA is a reliable multi-element analysis method for the direct quantitative analysis of solid samples. It possesses very high Au sensitivity and is minimally affected by matrix effects. It also allows avoiding losses and contamination during the pre-treatment stage because no digestion or pre-enrichment of the sample is required. Constantin used this technique to determine the gold contents of 82 geochemical reference materials in 2006 and 2009 [28,138] and compared them with the results obtained by other analytical techniques. Table 7 compares various analytical methods commonly used for the gold determination in rocks.

## 5. Conclusions and Perspectives

In this review, various sample digestion and combined preconcentration methods for the determination of ultra-low gold contents in rocks are summarized. Although some breakthroughs have been achieved in recent years, many important problems remain to be solved, owing to the heterogeneous distribution of gold in rocks, unknown forms of Au in different types of samples, and limited knowledge of the stability of gold compounds. These issues can also create promising opportunities in the study area; thus, future works in this field should focus on the following points:

1. In order to eliminate the nugget effect, the sample weight must be large enough. However, it leads to low digestion efficiency and requires the use of complex operating procedures. Therefore, additional studies must be performed to improve the sample representativeness.
2. Normally, the results obtained by the methods described above exhibit large deviations, which are often attributed to the nugget effect. However, homogeneous reference materials are required to confirm this conclusion.
3. Although FA can attack the entire rock sample, its relatively large reagent blank makes it difficult to determine the low gold content precisely. Wet acid digestion can solve this problem, but aqua regia may partially dissolve the rock samples. The desilication by HF is an effective process; however, it is inconvenient for the use in high-pressure ashers and Carius tubes and may cause a severe interference of ^181^Ta^16^O into ^197^Au determination when ICP-MS is utilized. In addition, the formation of insoluble fluoride compounds may also cause the loss of gold.
4. It should be noted that gold solutions must be analyzed as soon as possible after separation due to the instability of gold in both the HCl and thiourea media. In addition, the memory effect and instrument damage caused by their usage are normally large. Therefore, future research works may focus on the development of suitable media for gold elution and quantitation.

## Figures and Tables

**Table 1:** A summary of various FA digestion methods.

Sample Weight/g	Collector/Flux	Separation Technique	Detection Technique	LOD/ppb	Reference
10–30	Pb/Na_2_CO_3_, Na_2_B_4_O_7_, SiO_2_, flour(C)		ICP-MS	2	[38]
50	NiS/Na_2_CO_3_, Na_2_B_4_O_7_, CaF_2_		ICP-MS	0.023	[41]
15	NiS/Na_2_CO_3_, Na_2_B_4_O_7_, SiO_2_	Te coprecipitation	ICP-MS	1.69	[46]
15	NiS/Na_2_CO_3_, Na_2_B_4_O_7_, SiO_2_	Te coprecipitation	ICP-MS	0.484	[47]
20	NiS/Na_2_CO_3_, Na_2_B_4_O_7_, SiO_2_	duplicate NiS-FA and Te coprecipitation	ICP-MS	0.053	[48]
20	NiS/Na_2_CO_3_, Li_2_B_4_O_7_, SiO_2_	Te coprecipitation	ICP-MS	0.33	[49]
10	NiS/Na_2_CO_3_, Na_2_B_4_O_7_		INAA	2	[53]
10–15 50	NiS/Na_2_CO_3_, Na_2_B_4_O_7_ Pb/Na_2_CO_3_, NaOH, Na_2_B_4_O_7_, SiO_2_, C		UV-LA-ICP-MS FS-LA-ICP-MS	1.7 4	[54,60]

**Table 2:** A summary of various fusion digestion methods.

Sample Weight/g	Crucible	Flux	Separation Technique	Detection Technique	LOD/ppb	Reference
0.5	zirconium	Na_2_O_2_	Anion resin	GFAAS	-	[63]
1.0	graphite	Na_2_O_2_	Anion resin	USN-ICP-MS+NAA	ppt/-	[64]
1–20	zirconium	Na_2_O_2_/NaKCO_3_/KOH	Se-Te coprecipitation	ICP-MS	0.58	[65]
1–20 2	Corundum Corundum	Na_2_O_2_ Na_2_O_2_	Te coprecipitation Te coprecipitation	ICP-MS ICP-MS	0.007 0.32	[66,67]

**Table 3:** Preconcentration of Au by various precipitation methods.

Sample Weight/g	Digestion	Dissolution	Separation Technique	Detection Technique	LOD/ppb	Reference
2–5	Teflon beaker	HF + aq.reg.	Te coprecipitation	GFAAS	-	[81]
0.3–1.3	Teflon beaker	HNO_3_ + HF + HClO_4_ + HCl	Te coprecipitation	INAA	0.7	[82]
0.5–1.5	PTFE bomb	HNO_3_ + HF + aq.reg. + HClO_4_ + HCl	Se coprecipitation	GFAAS/TXRF	0.2/1.2	[26]
0.5	Borosilicate tube	HCl + HNO_3_	Hg coprecipitation	GFAAS	0.3	[83]

**Table 4:** Various methods for the preconcentration of Au by ion exchange resins.

Sample Weight/g	Digestion	Dissolution	Separation Technique	Detection Technique	LOD/ppb	Reference
2	-	HNO_3_ + HCl + HClO_4_ + HF	Anion exchange resin	GFAAS	0.2	[84]
5	Teflon beaker	HF + aq.reg. + HNO_3_ + HCl	Cation exchange resin	GFAAS	-	[85]
2	Carius tube/HPA-s	HCl + HNO_3_	Cation exchange resin	ICP-MS	-	[5]
0.25	Microwave digestion	aq.reg. + HF + HClO_4_	Chelating resin	FI-ICP-MS	1.2	[71]
0.05–1.5	Mild heating	HNO_3_ + HClO_4_ + HF	Chelating resin	ETV-ICP-AES	0.075	[87]
5–10	-	aq.reg.	Chelating sorbent	GFAAS	0.5	[88]

**Table 5:** Methods for the preconcentration of Au by solvent extraction/dispersive liquid-liquid microextraction.

Sample Weight/g	Digestion	Dissolution	Separation Technique	Detection Technique	LOD/ppb	Reference
0.1–2.0	Borosilicate beaker	HNO_3_ + HCl	MIBK	GFAAS	-	[89]
0.5–2.0	Teflon beaker	aq.reg. + HF	MIBK	GFAAS	-	[90]
1	PFTE beaker	HClO_4_ + HF + aq.reg.	diethyl ether + MIBK	GFAAS	0.13	[27]
10	Glass beaker	aq.reg.	MIBK	GFAAS	0.1	[91]
5–10	Borosilicate beaker	HBr + Br_2_	IBMK	GFAAS	15	[92]
10	Erlenmeyer flask	HCl + HNO_3_	IBMK	GFAAS	0.2	[93]
0.2	-	aq.reg.	DLLME	GFAAS	0.005	[95]
0.003–0.03	Eppednorf vail	HNO_3_ + HCl	DLLME	GFAAS	1.5	[97]
0.02	-	HNO_3_ + HCl + HF	DLLME	GFAAS	0.002	[98]

**Table 6:** Methods for the preconcentration of Au by PUF/extraction chromatography/novel solid absorbents.

Sample Weight/g	Digestion	Dissolution	Separation Technique	Detection Technique	LOD/ppb	Reference
10	Polypropylene beaker	aq.reg.	PUF	GFAAS	0.23	[115]
10–20	-	aq.reg.	MIBK-loaded PUF	GFAAS	-	[124]
4	Teflon pot	HNO_3_ + HF + HCl + aq.reg.	DIBK-loaded CG71 resin	ICP-MS	0.002	[36]
0.2	Microwave vessel	aq.reg.	Single granular carbon	GFAAS	0.9	[134]
0.5	Microwave vessel	aq.reg.	Modified carbon nanotubes	SS-HR-CS-GFAAS	0.002	[135]
10	PFA vessel	aq.reg.	hybrid adsorbent	GFAAS	0.008	[136]
5–10	Hot-plate	aq.reg.	magnetic nanoparticles	FI-column-GFAAS	0.16	[137]

**Table 7:** Various analytical methods commonly used for the gold determination in rocks.

Sample Weight/g	Dissolution	Separation Technique	Detection Technique	LOD/ppb	Reference
15	NiS/Na_2_CO_3_, Na_2_B_4_O_7_, SiO_2_	Te coprecipitation	ICP-MS	0.484	[47]
1–20	Na_2_O_2_	Te coprecipitation	ICP-MS	0.007	[66]
0.5–1.5	HNO_3_ + HF + aq.reg. + HClO_4_ + HCl	Se coprecipitation	GFAAS/TXRF	0.2/1.2	[26]
2	HCl + HNO_3_	Cation exchange resin	ICP-MS	-	[5]
0.02	HNO_3_ + HCl + HF	DLLME	GFAAS	0.002	[98]
10	aq.reg.	PUF	GFAAS	0.23	[115]
4	HNO_3_ + HF + HCl + aq.reg.	DIBK-loaded CG71 resin	ICP-MS	0.002	[36]
0.2	aq.reg.	Single granular carbon	GFAAS	0.9	[134]
5–10	aq.reg.	magnetic nanoparticles	FI-column-GFAAS	0.16	[137]
1–3	-	-	INAA	~0.1	[138]
